# Long-term effects of sitagliptin in patients with type 2 diabetes mellitus and hypertension: results from the PROLOGUE study

**DOI:** 10.18632/oncotarget.22959

**Published:** 2017-12-06

**Authors:** Ziliang Ye, Hui Li, Haili Lu, Qiang Su, Lang Li

**Affiliations:** ^1^ Department of Cardiology, The First Affiliated Hospital of Guangxi Medical University, Nanning, Guangxi, China; ^2^ Department of Orthodontics, The Affiliated Dental Hospital of Guangxi Medical University, Nanning, Guangxi, China; ^3^ Guangxi Medical University, Nanning, Guangxi, China

**Keywords:** dipeptidyl peptidase 4 inhibitors, sitagliptin, type 2 diabetes, hypertension, PROLOGUE study

## Abstract

**Background:**

The effects of sitagliptin in patients with type 2 diabetes mellitus and hypertension are unclear. Therefore, we evaluated the long-term effects of sitagliptin in those patients.

**Methods:**

In the PROLOGUE study, 365 patients were diagnosed as type 2 diabetes mellitus and hypertension, and 189 patients in the sitagliptin group, 176 patients in the conventional group. Fasting blood glucose (FBG), HbA1c, systolic pressure (SP), diastolic pressure (DP), serum urea nitrogen (BUN) and serum creatinine (SCR) were measured at the beginning of the study and after 12 and 24 months of treatment.

**Results:**

FBS and HbA1c levels were not significantly decreased after treatment [12 months: OR: -3.1, 95% CI (-11.3, 5.0); OR: 0.1, 95% CI (0.0, 0.3); 24 months: OR: -0.1, 95% CI (-9.1, 8.8); OR: 0.1, 95% CI (0.0, 0.3), respectively]. BP and DP levels were not significantly decreased after treatment (12 months: OR: 0.9, 95% CI (-2.8, 4.6); OR: 0.6, 95% CI (-2.0, 3.2); 24 months: OR: -0.5, 95% CI (-4.2, 3.1); OR: -1.6, 95% CI (-41, 0.9), respectively]. Furthermore, BUN and SCR levels were not significantly decreased after treatment (12 months: OR: 0.0, 95%CI (-1.2, 1.2); OR: 0.0, 95% CI (-0.1, 0.0); 24 months: OR: 0.4, 95% CI (-1.0, 1.8); OR: -80.8, 95% CI (-201.3, 39.8), respectively]. After adjusting for confounding factors, our results did not change.

**Conclusions:**

In our study, there was no evidence that treatment with sitagliptin can improve FBS, BP, DP, BUN or SCR in patients with type 2 diabetes mellitus and hypertension.

**Trial Registration:**

University Hospital Medical Information Network Clinical Trials Registry UMIN000004490.

## INTRODUCTION

With the development of the social economy, all kinds of pressure, the change in lifestyle and the aging population, the incidence of diabetes is increasing year by year. According to incomplete statistics, diabetes patients in China have exceeded the total number of diabetic patients in Europe and the United States, with China ranked second in the world [1, 2]. Diabetes has become a heavy burden of society and family life and to people’s health and quality of life. The current situation of the prevention and treatment of diabetes and their complications are serious.

Type 2 diabetes is a chronic, complex metabolic disease [3]. Its pathological mechanism is mainly that the molecular structure of insulin is abnormal or there is an absolute deficiency of insulin secretion, which can cause blood sugar to be unable to enter cells, and sustained high blood glucose levels can cause plasma osmotic pressure to increase significantly, resulting in cellular environment change [4]. Meanwhile, type 2 diabetes can cause disorders of glucose, lipid and protein metabolism, which can result in complications of the heart and peripheral vascular system (such as coronary heart disease and hypertension), endocrine and metabolic system (such as ketoacidosis and lactic acidosis), and others [5, 6]. Among them, type 2 diabetes mellitus complicated with hypertension is a common clinical disease [7, 8]. Studies have shown that for patients with type 2 diabetes and hypertension, the control of blood glucose and blood pressure is more difficult than that of single disease [8, 9]. Moreover, the combination of diseases can damage blood vessels and renal function and increase the risk of vascular disease and renal failure.

Sitagliptin [10], a dipeptidyl peptidase-4 (DPP-4) inhibitor, is an antihyperglycemic drug that stimulate insulin release from pancreaticβ-cells by sparing incretin hormones. The mechanism of sitagliptin is different from the usual common oral hypoglycemic drugs, and it acts mainly through inhibiting the activity of DPP-4 to prolong the duration of GLP-1 to achieve steady blood sugar. In addition, sitagliptin can protect incretin activity, stimulate islet B-cell regeneration, improve glucose tolerance and insulin sensitivity, and delay the occurrence of diabetes [11].

However, whether sitagliptin is advantageous to the prognosis of type 2 diabetes patients with hypertension, the conclusion is still controversial. Ogawa S [12] conducted a study lasting six months and determined that sitagliptin can lower SP without reducing body mass index, independent of blood glucose reduction. However, Yuasa S [13] carried out a retrospective cohort study in 454 patients with type 2 diabetes and showed that BP was slightly but significantly reduced after 6 months of sitagliptin therapy, indicating that sitagliptin has pleiotropic effects, including an antihypertensive effect.

The PROLOGUE study [10] was a prospective multicenter study conducted to evaluate the inhibitory effect of a DPP-4 inhibitor on the progression of atherosclerosis based on carotid artery intima-media thickness (IMT) assessed by ultrasonography over a 2-year follow-up period. In the PROLOGUE study, FBS, HbA1c, BP, DP, BUN and SCR were measured in all patients. Thus, we carried out this study as a sub-analysis of the PROLOGUE study to evaluate the long-term effect of sitagliptin in patients with type 2 diabetes mellitus and hypertension.

## RESULTS

### Baseline clinical characteristics

Table 1 shows the baseline clinical characteristics of all patients and the effects of each treatment on baseline parameters in the sitagliptin group and the conventional group. Of the 365 patients, 248 (67.94%) were men and 117 (32.06%) were women. 262 (71.78%) had dyslipidemia, 30 (8.21%) had kidney disease, 17 (4.66%) had liver disease, 52 (14.25%) had a cerebrovascular disorder, 212 (58.08%) had cardiovascular disorder, 42 (11.78%) had cerebral infarction, 3 (0.82%) had cerebral hemorrhage, 5 (1.37%) had transient ischemic attacks, 3 (0.82%) had cerebrovascular disorder, 79 (21.64%) had myocardial infarction, 52 (14.24%) had arrhythmia, 103 (28.22%) had percutaneous coronary intervention, 28 (7.67%) had heart failure, 86 (23.56%) had cardiovascular disorder, 91 (24.93%) had a smoking habit, 40 (10.96%) had a drinking habit, and 27 (7.40%) had a coronary artery bypass graft. There was no significant difference in any of the variables between the two groups, including age, body height, body weight, DBP, SBP, creatinine, heart rate, uric acid, total cholesterol, fasting plasma glucose, HDL, blood urea nitrogen, HBA1C, cystatin-C, triglyceride, waist circumferences, small dense LDL, high molecular weight adiponectin and insulin levels.

**Table 1:** Baseline clinical characteristics

Allocation	Male			Female		
	Sitagliptin	Conventional	P value	Sitagliptin	Conventional	P value
N	128	120		61	56	
Age (year)	68.3±9.5	68.5±9.0	0.890	71.4±9.6	72.1±8.8	0.684
Body height (cm)	164.5±6.5	165.0±5.6	0.534	151.0 ± 5.1	149.4±5.9	0.127
Body weight (kg)	69.7±11.1	69.1±11.2	0.673	58.4±13.9	57.8±10.4	0.809
Diastolic pressure (mm Hg)	73.4±9.8	73.5±11.3	0.962	72.1±11.5	71.3±10.9	0.719
Systolic pressure (mm Hg)	130.7±15.4	128.9±15.2	0.390	131.5±14.1	136.2±18.2	0.117
Creatinine (μmol/l)	1.0±0.2	0.9±0.3	0.700	0.7±0.2	0.7±0.1	0.331
Heart rate (bpm)	70.5±11.7	70.7±11.7	0.917	72.5±12.7	73.5±11.9	0.675
Uric acid (mg/dl)	6.1±1.2	6.1±1.4	0.876	5.3±1.3	5.0±1.3	0.221
Total cholesterol (mmol/l)	172.7±31.0	171.0±31.9	0.675	176.1±29.9	187.3±29.7	0.050
Fasting plasma glucose (mmol/l)	139.8±36.9	137.2±34.0	0.579	136.1±41.7	129.9±39.7	0.422
High density lipoprotein (mmol/l)	49.0±14.3	50.1±14.4	0.570	55.6±12.0	55.9±14.8	0.908
Blood urea nitrogen (mg/dl)	17.1±5.5	17.1±5.5	0.998	16.8±5.1	16.2±3.9	0.444
HBA1C (percent)	6.9±0.6	6.9±0.5	0.857	7.0±0.7	7.0±0.6	0.925
Cystatin-C (mg/l)	1.0±0.3	1.0±0.4	0.916	1.0±0.3	0.9±0.2	0.055
Triglyceride (mg/dl)	148.6±91.0	147.5±91.6	0.928	112.7±40.9	135.8±76.7	0.048
Waist circumstances (cm)	91.6±10.0	90.9±9.9	0.587	87.5±12.3	89.3±12.0	0.439
Small dense low density lipoprotein (md/dl)	35.5±18.5	35.0±16.1	0.854	30.7±15.2	32.7±16.9	0.521
High molecular weight adiponectin (ug/ml)	4.1±4.0	4.2±4.3	0.908	5.4±4.6	6.5±6.8	0.313
Insulin (pmol/ml)	15.3±14.5	15.2±17.0	0.984	10.3±7.0	13.2±13.9	0.268
History of dyslipidemia			0.800			0.787
No	36 (29.3%)	36 (30.8%)		11 (18.0%)	11 (20.0%)	
Yes	87 (70.7%)	81 (69.2%)		50 (82.0%)	44 (80.0%)	
History of kidney disease			0.926			0.303
No	111 (90.2%)	106 (90.6%)		56 (91.8%)	53 (96.4%)	
Yes	12 (9.8%)	11 (9.4%)		5 (8.2%)	2 (3.6%)	
History of liver disease			0.129			0.053
No	119 (96.7%)	108 (92.3%)		57 (93.4%)	55 (100.0%)	
Yes	4 (3.3%)	9 (7.7%)		4 (6.6%)	0 (0.0%)	
History of cerebrovascular disorder			0.595			0.799
No	108 (87.8%)	100 (85.5%)		51 (83.6%)	45 (81.8%)	
Yes	15 (12.2%)	17 (14.5%)		10 (16.4%)	10 (18.2%)	
History of cardiovascular disorder			0.671			0.298
No	41 (33.3%)	36 (30.8%)		38 (62.3%)	29 (52.7%)	
Yes	82 (66.7%)	81 (69.2%)		23 (37.7%)	26 (47.3%)	
History of cerebral infarction			0.444			0.975
No	112 (91.1%)	103 (88.0%)		52 (85.2%)	47 (85.5%)	
Yes	11 (8.9%)	14 (12.0%)		9 (14.8%)	8 (14.5%)	
History of cerebral hemorrhage			0.591			0.340
No	121 (98.4%)	116 (99.1%)		60 (98.4%)	55 (100.0%)	
Yes	2 (1.6%)	1 (0.9%)		1 (1.6%)	0 (0.0%)	
History of transient ischemic attacks			0.960			0.290
No	121 (98.4%)	115 (98.3%)		61 (100.0%)	54 (98.2%)	
Yes	2 (1.6%)	2 (1.7%)		0 (0.0%)	1 (1.8%)	
History of cerebrovascular disorder			0.145			0.290
No	123 (100.0%)	115 (98.3%)		61 (100.0%)	54 (98.2%)	
Yes	0 (0.0%)	2 (1.7%)		0 (0.0%)	1 (1.8%)	
History of myocardial infarction			0.414			0.440
No	91 (74.0%)	81 (69.2%)		54 (88.5%)	51 (92.7%)	
Yes	32 (26.0%)	36 (30.8%)		7 (11.5%)	4 (7.3%)	
History of arrhythmia			0.862			0.674
No	103 (83.7%)	97 (82.9%)		54 (88.5%)	50 (90.9%)	
Yes	20 (16.3%)	20 (17.1%)		7 (11.5%)	5 (9.1%)	
History of percutaneous coronary intervention			0.338			0.456
No	87 (70.7%)	76 (65.0%)		49 (80.3%)	41 (74.5%)	
Yes	36 (29.3%)	41 (35.0%)		12 (19.7%)	14 (25.5%)	
History of heart failure			0.726			0.105
No	112 (91.1%)	108 (92.3%)		59 (96.7%)	49 (89.1%)	
Yes	11 (8.9%)	9 (7.7%)		2 (3.3%)	6 (10.9%)	
History of cardiovascular disorder			0.088			0.457
No	97 (78.9%)	81 (69.2%)		50 (82.0%)	42 (76.4%)	
Yes	26 (21.1%)	36 (30.8%)		11 (18.0%)	13 (23.6%)	
Smoking habit			0.272			0.088
No	49 (45.8%)	36 (35.6%)		41 (77.4%)	30 (66.7%)	
Yes	38 (35.5%)	46 (45.5%)		1 (1.9%)	6 (13.3%)	
Past	20 (18.7%)	19 (18.8%)		11 (20.8%)	9 (20.0%)	
Drinking habit			0.555			0.103
No	34 (32.1%)	34 (33.7%)		42 (77.8%)	33 (73.3%)	
Yes	21 (19.8%)	15 (14.9%)		0 (0.0%)	4 (8.9%)	
Past	41 (38.7%)	37 (36.6%)		2 (3.7%)	3 (6.7%)	
Unknown	10 (9.4%)	15 (14.9%)		10 (18.5%)	5 (11.1%)	
History of coronary artery bypass graft			0.746			0.209
No	111 (90.2%)	107 (91.5%)		57 (93.4%)	54 (98.2%)	
Yes	12 (9.8%)	10 (8.5%)		4 (6.6%)	1 (1.8%)	

Data are presented as number (percent) or mean±SD.

### FBS, HbA1c, BP, DP, BUN and SCR levesl after 12 months of treatment

Table 2 shows the FBS, HbA1c, BP, DP, BUN and SCR levels after 12 months of treatment. FBS and HbA1c levels were not significantly decreased after 12 months in the sitagliptin group compared to the conventional group [OR: -3.1, 95% CI (-11.3, 5.0), P=0.449; OR: 0.1, 95% CI (0.0, 0.3), P=0.166, respectively]. BP and DP levels were not significantly decreased after 12 and 24 months in the sitagliptin group compared to the conventional group [OR: 0.9, 95% CI (-2.8, 4.6), P=0.630; OR: 0.6, 95% CI (-2.0, 3.2), P=0.654, respectively]. BUN and SCR levels were not significantly decreased after 12 months in the sitagliptin group compared to the conventional group [OR: 0.0, 95% CI (-1.2, 1.2), P=0.983; OR: 0.0, 95% CI (-0.1, 0.0), P=0.131, respectively].

**Table 2:** FBS, HbA1c, BP, DP, BUN and SCR level after 12 months of treatment

	Male	Female	Total
fasting plasma glucose at 12M (mg/dL)			
Sitagliptin	0	0	0
Conventional	-3.9 (-13.0, 5.3) 0.408	-1.5 (-18.2, 15.3) 0.865	-3.1 (-11.3, 5.0) 0.449
HbA1c at 12M (%)			
Sitagliptin	0	0	0
Conventional	0.2 (0.0, 0.3) 0.075	0.0 (-0.3, 0.3) 0.906	0.1 (0.0, 0.3) 0.166
systolic blood pressure at 12M (mmHg)			
Sitagliptin	0	0	0
Conventional	-2.2 (-6.4, 2.1) 0.316	7.8 (0.7, 14.9) 0.034	0.9 (-2.8, 4.6) 0.630
diastolic blood pressureat 12M (mmHg)			
Sitagliptin	0	0	0
Conventional	-0.2 (-3.4, 3.0) 0.892	2.4 (-2.1, 6.9) 0.292	0.6 (-2.0, 3.2) 0.654
blood urea nitrogen at 12M (mg/dL)			
Sitagliptin	0	0	0
Conventional	0.3 (-1.2, 1.7) 0.737	-0.5 (-2.8, 1.7) 0.652	0.0 (-1.2, 1.2) 0.983
Creatinine at 12M (mg/gCre)			
Sitagliptin	0	0	0
Conventional	0.0 (-0.1, 0.0) 0.311	-0.1 (-0.1, 0.0) 0.192	0.0 (-0.1, 0.0) 0.131

Results are presented as OR (95% CI) P value.

### FBS, HbA1c, BP, DP, BUN and SCR levels after 24 months of treatment

Table 3 shows the FBS, HbA1c, BP, DP, BUN and SCR levels after 24 months of treatment. FBS and HbA1c levels were not significantly decreased after 24 months in the sitagliptin group compared to the conventional group [OR: -0.1, 95% CI (-9.1, -8.8), P=0.979; OR: 0.1, 95% CI (0.0, -0.3), P=0.064, respectively]. BP and DP levels were not significantly decreased after 24 months in the sitagliptin group compared to the conventional group [24 months: OR: -0.5, 95% CI (-4.2, -3.1), P=0.775; OR: -1.6, 95% CI (-41, -0.9), P=0204, respectively]. BUN and SCR levels were not significantly decreased after 24 months in the sitagliptin group compared to the conventional group [OR: 0.4, 95% CI (-1.0, 1.8), P=0.568; OR: -80.8, 95% CI (-201.3, -39.8), P=0.191, respectively].

**Table 3:** FBS, HbA1c, BP, DP, BUN and SCR level after 24 months of treatment

	Male	Female	Total
fasting plasma glucose at 24M (mg/dL)			
Sitagliptin	0	0	0
Conventional	-1.2 (-5.7, 3.2) 0.585	1.0 (-5.4, 7.4) 0.758	-0.5 (-4.2, 3.1) 0.775
HbA1c at 24M (%)			
Sitagliptin	0	0	0
Conventional	-1.0 (-3.9, 2.0) 0.526	-3.0 (-7.7, 1.6) 0.199	-1.6 (-4.1, 0.9) 0.204
systolic blood pressure at 24M (mmHg)			
Sitagliptin	0	0	0
Conventional	-4.3 (-7.7, -0.8) 0.017	0.2 (-4.6, 4.9) 0.948	-2.9 (-5.7, -0.1) 0.044
diastolic blood pressureat 24M (mmHg)			
Sitagliptin	0	0	0
Conventional	0.8 (-0.8, 2.5) 0.334	-0.5 (-3.2, 2.2) 0.726	0.4 (-1.0, 1.8) 0.568
blood urea nitrogen at 24M (mg/dL)			
Sitagliptin	0	0	0
Conventional	-0.8 (-11.8, 10.2) 0.886	1.4 (-14.1, 16.9) 0.861	-0.1 (-9.1, 8.8) 0.979
Creatinine at 24M (mg/gCre)			
Sitagliptin	0	0	0
Conventional	0.1 (-0.1, 0.3) 0.388	0.3 (0.0, 0.6) 0.053	0.1 (0.0, 0.3) 0.064
fasting plasma glucose at 24M (mg/dL)			
Sitagliptin	0	0	0
Conventional	-119.0 (-284.8, 46.8) 0.162	19.7 (-4.1, 43.4) 0.110	-80.8 (-201.3, 39.8) 0.191

Results are presented as OR (95% CI) P value.

### Subgroup analysis of FBS, HbA1c, BP, DP, BUN and SCR levels after 12 months of treatment

Table 4 shows the subgroup analysis of FBS, HbA1c, BP, DP, BUN and SCR levels after 12 months of treatment. After grouped by history of cardiovascular disorder, history of cerebral infarction, history of myocardial infarction, history of percutaneous coronary intervention and history of coronary artery bypass graft, we did not find that the FBS, HbA1c, BP, DP, BUN or SCR levels in the sitagliptin group were better than those of the conventional group (all P>0.05).

**Table 4:** Subgroup analysis of FBS, HbA1c, BP, DP, BUN and SCR level after 12 months of treatment

	N	FBS at 12M (mg/dL)	HbA1c at 12M (%)	SBP at 12M (mmHg)	DBP at 12M (mmHg)	BUN at 12M (mg/dL)	Creatinine at 12M (mg/gCre)
History of cardiovascular disorder							
No	144	-4.6 (-17.4, 8.3) 0.487	0.1 (-0.1, 0.4) 0.347	4.7 (-0.2, 9.6) 0.065	2.2 (-1.3, 5.6) 0.217	0.2 (-1.7, 2.0) 0.870	0.0 (-0.1, 0.1) 0.534
Yes	212	-2.0 (-12.6, 8.5) 0.706	0.1 (-0.1, 0.3) 0.330	-1.6 (-6.8, 3.6) 0.547	-0.1 (-3.9, 3.6) 0.942	-0.2 (-1.8, 1.4) 0.800	0.0 (-0.1, 0.0) 0.260
History of cerebral infarction							
No	314	-3.1 (-11.9, 5.6) 0.485	0.1 (0.0, 0.3) 0.137	2.0 (-1.9, 5.8) 0.318	1.1 (-1.8, 3.9) 0.470	0.1 (-1.3, 1.4) 0.925	0.0 (-0.1, 0.0) 0.312
Yes	42	0.6 (-20.8, 22.0) 0.958	0.0 (-0.4, 0.4) 0.983	-9.9 (-21.2, 1.3) 0.092	-2.1 (-8.5, 4.2) 0.514	-0.3 (-3.5, 3.0) 0.868	0.0 (-0.1, 0.2) 0.906
History of myocardial infarction							
No	277	-2.9 (-12.6, 6.8) 0.555	0.1 (0.0, 0.3) 0.145	1.1 (-3.0, 5.2) 0.595	0.4 (-2.6, 3.3) 0.798	-0.2 (-1.6, 1.2) 0.790	0.0 (-0.1, 0.0) 0.215
Yes	79	-2.2 (-16.2, 11.8) 0.755	0.0 (-0.2, 0.2) 0.928	0.1 (-8.2, 8.4) 0.986	1.9 (-3.8, 7.7) 0.510	0.6 (-1.7, 3.0) 0.607	0.0 (-0.1, 0.1) 0.696
History of percutaneous coronary intervention							
No	253	-4.2 (-14.2, 5.8) 0.414	0.1 (-0.1, 0.3) 0.171	2.9 (-1.4, 7.1) 0.185	1.5 (-1.6, 4.6) 0.345	0.2 (-1.3, 1.7) 0.806	-0.1 (-0.1, 0.0) 0.080
Yes	103	2.3 (-11.6, 16.3) 0.742	0.1 (-0.2, 0.4) 0.704	-4.7 (-12.2, 2.8) 0.225	-1.2 (-6.2, 3.9) 0.650	-0.4 (-2.7, 1.8) 0.708	0.0 (0.0, 0.1) 0.316
History of coronary artery bypass graft							
No	329	-3.8 (-12.4, 4.9) 0.395	0.1 (-0.1, 0.3) 0.264	1.0 (-2.8, 4.7) 0.616	0.8 (-1.8, 3.4) 0.530	0.2 (-1.1, 1.4) 0.789	0.8 (-1.8, 3.4) 0.530
Yes	27	2.7 (-16.6, 22.0) 0.789	0.2 (-0.1, 0.6) 0.155	-2.7 (-21.3, 15.8) 0.775	-1.0 (-14.7, 12.6) 0.883	-1.0 (-5.7, 3.7) 0.676	-1.0 (-14.7, 12.6) 0.883

Results are presented as OR (95% CI) P value.

FBS: fasting plasma glucose; SBP: systolic blood pressure; DBP: diastolic blood pressure; BUN: Blood urea nitrogen.

### Subgroup analysis of FBS, HbA1c, BP, DP, BUN and SCR levels after 24 months of treatment

Table 5 shows the subgroup analysis of FBS, HbA1c, BP, DP, BUN and SCR levels after 24 months of treatment. After grouped by history of cardiovascular disorder, history of cerebral infarction, history of myocardial infarction, history of percutaneous coronary intervention and history of coronary artery bypass graft, we did not find that the FBS, HbA1c, BP, DP, BUN and SCR levels in the sitagliptin group was better than those of the conventional group (all P>0.05).

**Table 5:** Subgroup analysis of FBS, HbA1c, BP, DP, BUN and SCR level after 24 months of treatment

	N	FBS at 24M (mg/dL)	HbA1c at 24M (%)	SBP at 24M (mmHg)	DBP at 24M (mmHg)	BUN at 24M (mg/dL)	Creatinine at 24M (mg/gCre)
History of cardiovascular disorder							
No	144	3.7 (-9.2, 16.5) 0.575	0.1 (0.0, 0.3) 0.134	2.0 (-3.0, 7.0) 0.427	0.0 (-3.7, 3.8) 0.985	0.0 (-0.1, 0.1) 0.954	0.0 (-2.2, 2.2) 0.979
Yes	212	-2.9 (-15.2, 9.5) 0.650	0.1 (-0.1, 0.4) 0.266	-2.1 (-7.2, 3.0) 0.429	-2.3 (-5.6, 1.0) 0.170	0.0 (-0.1, 0.1) 0.614	0.6 (-1.3, 2.4) 0.546
History of cerebral infarction							
No	314	-0.6 (-10.4, 9.2) 0.905	0.2 (0.0, 0.3) 0.035	0.1 (-3.8, 4.0) 0.975	-1.5 (-4.2, 1.3) 0.291	0.0 (-0.1, 0.1) 0.784	0.5 (-1.0, 2.1) 0.496
Yes	42	7.1 (-13.8, 27.9) 0.512	0.0 (-0.7, 0.6) 0.886	-6.8 (-17.3, 3.7) 0.214	-2.0 (-7.3, 3.4) 0.479	0.1 (-0.1, 0.2) 0.460	-0.5 (-3.6, 2.6) 0.752
History of myocardial infarction							
No	277	-0.1 (-10.3, 10.0) 0.982	0.2 (0.0, 0.3) 0.098	0.5 (-3.5, 4.4) 0.806	-1.3 (-4.2, 1.6) 0.390	0.0 (-0.1, 0.1) 0.654	-0.1 (-1.8, 1.6) 0.893
Yes	79	0.0 (-19.3, 19.2) 0.998	0.1 (-0.2, 0.4) 0.420	-4.0 (-12.8, 4.8) 0.375	-2.3 (-7.0, 2.3) 0.325	0.0 (-0.1, 0.2) 0.423	2.1 (-0.3, 4.5) 0.092
History of percutaneous coronary intervention							
No	253	-0.5 (-11.7, 10.7) 0.931	0.1 (0.0, 0.3) 0.146	1.3 (-2.7, 5.4) 0.523	-0.6 (-3.6, 2.4) 0.683	0.0 (-0.1, 0.1) 0.508	0.4 (-1.4, 2.2) 0.665
Yes	103	3.1 (-11.9, 18.0) 0.688	0.1 (-0.1, 0.4) 0.307	-5.8 (-13.7, 2.1) 0.156	-2.9 (-7.1, 1.4) 0.188	0.0 (-0.1, 0.2) 0.438	0.4 (-1.8, 2.6) 0.726
History of coronary artery bypass graft							
No	329	-0.4 (-10.0, 9.3) 0.941	0.1 (0.0, 0.3) 0.122	-1.1 (-4.9, 2.6) 0.557	-1.9 (-4.5, 0.6) 0.143	0.0 (-0.1, 0.1) 0.846	0.5 (-0.9, 2.0) 0.480
Yes	27	4.9 (-17.2, 27.1) 0.667	0.2 (-0.2, 0.6) 0.266	4.3 (-11.0, 19.7) 0.585	2.7 (-5.5, 10.9) 0.524	-0.1 (-0.3, 0.1) 0.402	-0.4 (-5.5, 4.6) 0.866

Results are presented as OR (95% CI) P value.

FBS: fasting plasma glucose; SBP: systolic blood pressure; DBP: diastolic blood pressure; BUN: Blood urea nitrogen.

### Multivariate regression analysis of FBS, HbA1c, BP, DP, BUN and SCR levels after 12 months of treatment

Table 6 shows the multivariate regression analysis of FBS, HbA1c, BP, DP, BUN and SCR levels after 12 months of treatment. First, the non-adjusted model was not adjusted. Second, the adjust I model was adjusted for age, body height, body weight, DBP, SBP, creatinine, heart rate, uric acid, total cholesterol, fasting plasma glucose, HDL, blood urea nitrogen, HBA1C, cystatin-C, triglyceride, waist circumferences, small dense LDL, high molecular weight adiponectin and insulin. Third, the adjust II model was adjusted for age, body height, body weight, DBP, SBP, creatinine, heart rate, uric acid, total cholesterol, fasting plasma glucose, HDL, blood urea nitrogen, HBA1C, cystatin-C, triglyceride, waist circumferences, small dense LDL, high molecular weight adiponectin, insulin, history of dyslipidemia, history of kidney disease, history of liver disease, history of cerebrovascular disorder, history of cardiovascular disorder, history of cerebral infarction, history of cerebral hemorrhage, history of transient ischemic attacks, history of cerebrovascular disorder, history of myocardial infarction, history of arrhythmia, history of percutaneous coronary intervention, history of heart failure, history of cardiovascular disorder, smoking habit, drinking habit, and history of coronary artery bypass graft. However, we did not find any differences between the two groups except for in systolic blood pressure at 12 months in the non-adjusted and adjust I analyses in females (P<0.05).

**Table 6:** Multivariate regression analysis of FBS, HbA1c, BP, DP, BUN and SCR level after 12 months of treatment

Exposure	Male	Female	Total
Systolic blood pressure at 12M			
Non-adjusted			
Sitagliptin	0	0	0
Conventional	-2.2 (-6.4, 2.1) 0.316	7.8 (0.7, 14.9) 0.034	0.9 (-2.8, 4.6) 0.630
Adjust I			
Sitagliptin	0	0	0
Conventional	-2.7 (-7.3, 2.0) 0.263	8.0 (0.2, 15.8) 0.049	-0.6 (-4.6, 3.4) 0.766
Adjust II			
Sitagliptin	0	0	0
Conventional	-3.3 (-8.0, 1.5) 0.178	6.5 (-1.6, 14.5) 0.119	-1.0 (-5.1, 3.1) 0.633
Diastolic blood pressure at 12M			
Non-adjusted			
Sitagliptin	0	0	0
Conventional	-0.2 (-3.4, 3.0) 0.892	2.4 (-2.1, 6.9) 0.292	0.6 (-2.0, 3.2) 0.654
Adjust I			
Sitagliptin	0	0	0
Conventional	0.1 (-3.3, 3.5) 0.948	2.4 (-2.8, 7.7) 0.362	0.4 (-2.3, 3.2) 0.755
Adjust II			
Sitagliptin	0	0	0
Conventional	-0.2 (-3.6, 3.2) 0.925	2.5 (-3.0, 7.9) 0.380	0.3 (-2.5, 3.1) 0.847
Pulse rate at 12M			
Non-adjusted			
Sitagliptin	0	0	0
Conventional	0.7 (-2.2, 3.6) 0.627	2.5 (-2.3, 7.4) 0.311	1.3 (-1.2, 3.8) 0.321
Adjust I			
Sitagliptin	0	0	0
Conventional	0.6 (-2.7, 3.8) 0.733	5.2 (-1.1, 11.5) 0.110	1.5 (-1.3, 4.3) 0.301
Adjust II			
Sitagliptin	0	0	0
Conventional	0.3 (-3.1, 3.6) 0.876	5.1 (-1.5, 11.8) 0.137	1.4 (-1.5, 4.3) 0.361
HbA1c at 12M			
Non-adjusted			
Sitagliptin	0	0	0
Conventional	0.2 (0.0, 0.3) 0.075	0.0 (-0.3, 0.3) 0.906	0.1 (0.0, 0.3) 0.166
Adjust I			
Sitagliptin	0	0	0
Conventional	0.2 (0.0, 0.4) 0.043	0.0 (-0.3, 0.3) 0.899	0.1 (0.0, 0.3) 0.120
Adjust II			
Sitagliptin	0	0	0
Conventional	0.2 (0.0, 0.4) 0.032	0.0 (-0.4, 0.3) 0.856	0.1 (0.0, 0.3) 0.140
Blood urea nitrogen at 12M			
Non-adjusted			
Sitagliptin	0	0	0
Conventional	0.3 (-1.2, 1.7) 0.737	-0.5 (-2.8, 1.7) 0.652	0.0 (-1.2, 1.2) 0.983
Adjust I			
Sitagliptin	0	0	0
Conventional	0.7 (-0.8, 2.2) 0.389	-0.8 (-3.5, 1.8) 0.543	0.1 (-1.1, 1.4) 0.819
Adjust II			
Sitagliptin	0	0	0
Conventional	0.7 (-0.8, 2.2) 0.373	-0.3 (-3.1, 2.4) 0.808	0.2 (-1.1, 1.5) 0.736
Fasting plasma glucose at 12M			
Non-adjusted			
Sitagliptin	0	0	0
Conventional	-3.9 (-13.0, 5.3) 0.408	-1.5 (-18.2, 15.3) 0.865	-3.1 (-11.3, 5.0) 0.449
Adjust I			
Sitagliptin	0	0	0
Conventional	-5.2 (-15.4, 5.0) 0.322	-1.4 (-19.6, 16.8) 0.881	-3.5 (-12.2, 5.1) 0.420
Adjust II			
Sitagliptin	0	0	0
Conventional	-4.9 (-15.2, 5.4) 0.354	-3.3 (-22.8, 16.1) 0.738	-4.3 (-13.0, 4.4) 0.332
Urinary albumin/creatinine ratio at 12M			
Non-adjusted			
Sitagliptin	0	0	0
Conventional	0.0 (-0.1, 0.0) 0.311	-0.1 (-0.1, 0.0) 0.192	0.0 (-0.1, 0.0) 0.131
Adjust I			
Sitagliptin	0	0	0
Conventional	0.0 (-0.1, 0.0) 0.331	-0.1 (-0.2, 0.0) 0.069	0.0 (-0.1, 0.0) 0.111
Adjust II			
Sitagliptin	0	0	0
Conventional	0.0 (-0.1, 0.0) 0.273	-0.1 (-0.2, 0.0) 0.124	0.0 (-0.1, 0.0) 0.073

Results are presented as OR (95% CI) P value.

Non-adjusted model adjust for: None.

Adjust I model adjust for: age, body height, body weight, DBP, SBP, creatinine, heart rate, uric acid, total cholesterol, fasting plasma glucose, HDL, blood urea nitrogen, HBA1C, cystatin-C, triglyceride, waist circumstances, small dense LDL, high molecular weight adiponectin and insulin.

Adjust II model adjust for: age, body height, body weight, DBP, SBP, creatinine, heart rate, uric acid, total cholesterol, fasting plasma glucose, HDL, blood urea nitrogen, HBA1C, cystatin-C, triglyceride, waist circumstances, small dense LDL, high molecular weight adiponectin, insulin, history of dyslipidemia, history of kidney disease, history of liver disease, history of cerebrovascular disorder, history of cardiovascular disorder, history of cerebral infarction, history of cerebral hemorrhage, history of transient ischemic attacks, history of cerebrovascular disorder, history of myocardial infarction, history of arrhythmia, history of percutaneous coronary intervention, history of heart failure, history of cardiovascular disorder, smoking habit, drinking habit, history of coronary artery bypass graft.

### Multivariate regression analysis of FBS, HbA1c, BP, DP, BUN and SCR levels after 24 months of treatment

Table 7 shows the multivariate regression analysis of FBS, HbA1c, BP, DP, BUN and SCR levels after 24 months of treatment. First, the non-adjusted model was not adjusted. Second, the adjust I model was adjusted for age, body height, body weight, DBP, SBP, creatinine, heart rate, uric acid, total cholesterol, fasting plasma glucose, HDL, blood urea nitrogen, HBA1C, cystatin-C, triglyceride, waist circumferences, small dense LDL, high molecular weight adiponectin and insulin levels. Third, the adjust II model was adjusted for age, body height, body weight, DBP, SBP, creatinine, heart rate, uric acid, total cholesterol, fasting plasma glucose, HDL, blood urea nitrogen, HBA1C, cystatin-C, triglyceride, waist circumferences, small dense LDL, high molecular weight adiponectin, insulin, history of dyslipidemia, history of kidney disease, history of liver disease, history of cerebrovascular disorder, history of cardiovascular disorder, history of cerebral infarction, history of cerebral hemorrhage, history of transient ischemic attacks, history of cerebrovascular disorder, history of myocardial infarction, history of arrhythmia, history of percutaneous coronary intervention, history of heart failure, history of cardiovascular disorder, smoking habit, drinking habit, and history of coronary artery bypass graft. However, we did not find any differences between the two groups (all P>0.05).

**Table 7:** Multivariate regression analysis of FBS, HbA1c, BP, DP, BUN and SCR level after 24 months of treatment

Exposure	Male	Female	Total
Systolic blood pressure at 24M			
Non-adjusted			
Sitagliptin	0	0	0
Conventional	-1.2 (-5.7, 3.2) 0.585	1.0 (-5.4, 7.4) 0.758	-0.5 (-4.2, 3.1) 0.775
Adjust I			
Sitagliptin	0	0	0
Conventional	-1.0 (-5.8, 3.7) 0.671	3.7 (-4.0, 11.4) 0.349	-0.7 (-4.7, 3.2) 0.714
Adjust II			
Sitagliptin	0	0	0
Conventional	-1.2 (-6.0, 3.6) 0.614	3.6 (-4.3, 11.5) 0.373	-1.0 (-5.0, 3.0) 0.626
Diastolic blood pressure at 24M			
Non-adjusted			
Sitagliptin	0	0	0
Conventional	-1.0 (-3.9, 2.0) 0.526	-3.0 (-7.7, 1.6) 0.199	-1.6 (-4.1, 0.9) 0.204
Adjust I			
Sitagliptin	0	0	0
Conventional	-1.4 (-4.4, 1.7) 0.386	-3.4 (-8.6, 1.8) 0.203	-2.2 (-4.8, 0.3) 0.089
Adjust II			
Sitagliptin	0	0	0
Conventional	-1.3 (-4.3, 1.8) 0.421	-3.3 (-8.7, 2.0) 0.222	-2.1 (-4.7, 0.5) 0.115
Pulse rate at 24M			
Non-adjusted			
Sitagliptin	0	0	0
Conventional	-4.3 (-7.7, -0.8) 0.017	0.2 (-4.6, 4.9) 0.948	-2.9 (-5.7, -0.1) 0.044
Adjust I			
Sitagliptin	0	0	0
Conventional	-3.8 (-7.5, -0.1) 0.048	4.3 (-1.5, 10.0) 0.153	-2.5 (-5.5, 0.6) 0.112
Adjust II			
Sitagliptin	0	0	0
Conventional	-3.7 (-7.4, 0.1) 0.055	4.5 (-1.4, 10.4) 0.140	-2.3 (-5.4, 0.8) 0.145
HbA1c at 24M			
Non-adjusted			
Sitagliptin	0	0	0
Conventional	0.8 (-0.8, 2.5) 0.334	-0.5 (-3.2, 2.2) 0.726	0.4 (-1.0, 1.8) 0.568
Adjust I			
Sitagliptin	0	0	0
Conventional	1.3 (-0.4, 2.9) 0.127	-0.6 (-3.8, 2.7) 0.733	0.5 (-1.0, 2.0) 0.492
Adjust II			
Sitagliptin	0	0	0
Conventional	1.3 (-0.4, 3.0) 0.128	-0.2 (-3.5, 3.1) 0.909	0.6 (-0.9, 2.1) 0.447
Blood urea nitrogen at 24M			
Non-adjusted			
Sitagliptin	0	0	0
Conventional	-0.8 (-11.8, 10.2) 0.886	1.4 (-14.1, 16.9) 0.861	-0.1 (-9.1, 8.8) 0.979
Adjust I			
Sitagliptin	0	0	0
Conventional	1.9 (-9.9, 13.7) 0.752	-1.4 (-17.0, 14.2) 0.863	0.8 (-8.4, 9.9) 0.867
Adjust II			
Sitagliptin	0	0	0
Conventional	2.5 (-9.3, 14.4) 0.677	-1.4 (-17.5, 14.6) 0.860	1.4 (-7.8, 10.6) 0.768
Fasting plasma glucose at 24M			
Non-adjusted			
Sitagliptin	0	0	0
Conventional	0.1 (-0.1, 0.3) 0.388	0.3 (0.0, 0.6) 0.053	0.1 (0.0, 0.3) 0.064
Adjust I			
Sitagliptin	0	0	0
Conventional	0.1 (-0.1, 0.3) 0.208	0.2 (-0.1, 0.5) 0.175	0.2 (0.0, 0.3) 0.066
Adjust II			
Sitagliptin	0	0	0
Conventional	0.1 (-0.1, 0.3) 0.231	0.2 (-0.1, 0.5) 0.249	0.1 (0.0, 0.3) 0.089
Urinary albumin/creatinine ratio at 24M			
Non-adjusted			
Sitagliptin	0	0	0
Conventional	0.0 (-0.1, 0.1) 0.818	0.0 (-0.1, 0.1) 0.428	0.0 (-0.1, 0.0) 0.596
Adjust I			
Sitagliptin	0	0	0
Conventional	0.0 (-0.1, 0.1) 0.882	-0.1 (-0.2, 0.0) 0.232	0.0 (-0.1, 0.0) 0.548
Adjust II			
Sitagliptin	0	0	0
Conventional	0.0 (-0.1, 0.1) 0.913	-0.1 (-0.2, 0.0) 0.209	0.0 (-0.1, 0.0) 0.508

Results are presented as OR (95% CI) P value.

Non-adjusted model adjust for: None.

Adjust I model adjust for: age, body height, body weight, DBP, SBP, creatinine, heart rate, uric acid, total cholesterol, fasting plasma glucose, HDL, blood urea nitrogen, HBA1C, cystatin-C, triglyceride, waist circumstances, small dense LDL, high molecular weight adiponectin and insulin.

Adjust II model adjust for: age, body height, body weight, DBP, SBP, creatinine, heart rate, uric acid, total cholesterol, fasting plasma glucose, HDL, blood urea nitrogen, HBA1C, cystatin-C, triglyceride, waist circumstances, small dense LDL, high molecular weight adiponectin, insulin, history of dyslipidemia, history of kidney disease, history of liver disease, history of cerebrovascular disorder, history of cardiovascular disorder, history of cerebral infarction, history of cerebral hemorrhage, history of transient ischemic attacks, history of cerebrovascular disorder, history of myocardial infarction, history of arrhythmia, history of percutaneous coronary intervention, history of heart failure, history of cardiovascular disorder, smoking habit, drinking habit, history of coronary artery bypass graft.

## DISCUSSION

To our knowledge, the PROLOGUE study is the largest trial to investigate whether DPP-4 inhibitors (e.g., sitagliptin) slow the progression of carotid IMT in participants with T2DM. The major finding of our study is that there was no evidence that treatment with sitagliptin can improve fasting glucose, HbA1c, systolic pressure or diastolic pressure in type 2 diabetes patients complicated with hypertension during a 2-year study period. In addition, for patients with type 2 diabetes complicated with hypertension, the kidney is one of the most easily damaged organs, which can lead to renal insufficiency and even renal failure. The PROLOGUE study followed up the patients for two years, but our result did not find that sitagliptin has a protective effect on renal function of patients with type 2 diabetes complicated with hypertension, as the changes in serum urea nitrogen and serum creatinine levels were similar to the conventional group. These results may suggest that sitagliptin failed to inhibit fasting glucose, HbA1c, blood pressure and kidney progression relative to the conventional therapy in patients with type 2 diabetes complicated with hypertension, despite its glucose-lowering effect in patients with type 2 diabetes.

It is well known that, with the growth of age and the acceleration of population aging all over the world, the lifetime risk of cardiovascular disease in patients with type 2 diabetes is approximately 67-78%[14]. Management of patients with type 2 diabetes should not only aim to control glycemia but also include the modification of cardiovascular risk factors [15]. Research results showed that hypertension, including essential hypertension and secondary hypertension, affects approximately two-thirds of patients with type 2 diabetes and is an important contributing factor to cardiovascular complications [16]. Lowering blood pressure, including systolic and diastolic blood pressure, has been shown to reduce cardiovascular events in patients with type 2 diabetes and to exert a renoprotective effect [17, 18]. In 2003, the Joint National Committee (JNC) guidelines recommended a targets for systolic BP (SBP) of 130 mmHg and diastolic BP (DBP) of 80 mmHg in patients with type 2 diabetes complicated with hypertension [19]. However, the guidelines were recently updated in 2014 to recommend a target BP of 140/90 mmHg in those patients [20]. Therefore, combination therapy is often updated because blood pressure is difficult to control in patients with type 2 diabetes complicated with hypertension [10].

Sitagliptin, an orally administered dipeptidyl peptidase 4 (DPP-4) inhibitor, prolongs the action of incretin hormones, by inhibiting their breakdown [21]. This improves glycemic control in patients with type 2 diabetes, primarily by suppressing glucagon levels and increasing endogenous insulin secretion. So far, a great deal of results have shown that sitagliptin can significantly improve blood glucose [22] without increasing the risk of fractures [23] or heart failure [24] in patients with type 2 diabetes mellitus. Josse RG [23] et al. conducted a study to assess the association between sitagliptin use and the risk of fractures in type 2 diabetes patients, and 14 671 participants were randomized to sitagliptin (n=7332) or placebo (n=7339) in a double-blind trial. They noted that sitagliptin, compared with placebo, was not associated with a higher fracture risk [adjusted HR 1.03, P =0.745], with major osteoporotic fractures or with hip fractures. Ferreira J C A [25] et al. carried out a trial to evaluate the efficacy and safety of sitagliptin versus glipizide in patients with type 2 diabetes and moderate-to-severe chronic renal insufficiency. Patients (n=426) were randomized 1:1 to sitagliptin or glipizide, and the results showed that in patients with T2DM and chronic renal insufficiency, sitagliptin and glipizide provided similar A1C-lowering efficacy. However, sitagliptin was generally well-tolerated, with a lower risk of hypoglycemia and weight loss versus weight gain relative to glipizide. All these studies have shown that sitagliptin can significantly improve the prognosis of patients with type 2 diabetes. Nevertheless, studies regarding the effect of sitagliptin on patients with type 2 diabetes complicated with hypertension are scarce. In our study, 365 patients with type 2 diabetes complicated with hypertension were recruited, and our results showed that sitagliptin, compared with conventional treatment, cannot significant improve fasting glucose, HbA1c, systolic pressure, diastolic pressure and renal function in type 2 diabetes patients complicated with hypertension during a 2-year study period (all P>0.05). In addition, compared with conventional treatment, sitagliptin does not have the renal protective effects in those patients. Based on this result, we conducted a sensitivity analysis based on gender, but the results did not change significantly. Our results were different from those of Susumu Ogawa et al. In 2011, Susumu Ogawa [12] conducted a study to estimate whether sitagliptin can decrease systolic blood pressure in Japanese hypertensive patients with type 2 diabetes, and their findings suggest that sitagliptin lowers SBP without reducing BMI, independent of the blood glucose reduction. The hypotensive effect is apparent with the alternate-day regimen of sitagliptin at a lower dose compared to the everyday medication. Chinese scholars [26] conducted a systematic review and meta-analysis to evaluate the effects of dipeptidyl peptidase-4 inhibitors on blood pressure in patients with type 2 diabetes, and fifteen trials involving 5636 participants were identified. In addition, the results showed that compared with placebo or nontreatment, DPP-4 inhibitors showed BP-lowering effects for both SBP and DBP. However, no significant differences of associated BP improvement were found between DPP-4 inhibitors and GLP-1 RAs or other antidiabetic medications.

To date, mechanisms related to the antihypertensive effects of DPP4 therapies have not been fully elucidated and some evidence remains inconclusive [27]. Previous studies have suggested complex possible mechanisms, and separate mechanisms may play a role in mediating the BP-lowering effects of DPP-4 inhibitors [10]. First, weight loss and improved glycemic control have been indicated to not correlate with the BP-lowering effects. Second, some studies showed that stimulation of natriuresis is one of the core parts contributing to the antihypertensive effects of DPP-4 inhibitors, and vasodilation through GLP-1 receptor-dependent and independent pathways was also considered to be involved in the process. Third, DPP-4 cleaves a number of peptides besides GLP-1, many of which are vasoactive and could be classified into vasodilators and vasoconstrictors in respect of their actions on blood vessels. For example, as substrates of DPP-4, neuropeptide Y 1 (NPY1) and substance P might be related to the BP elevation accompanied with the combined inhibition of angiotensin converting enzyme (ACE) and DPP-4 [28].

On the other hand, the mechanism of DPP-4 inhibitors, such as sitagliptin, on the protective effect of renal function in type 2 diabetes patients complicated with hypertension remains unclear [29, 30]. Incretin-based agents may exert a beneficial effect in preventing diabetic complications beyond their metabolic effects. Preclinical data have shown that vildagliptin, a DPP-4 inhibitor, can prevent peripheral nerve degeneration in a diabetes-induced animal model. Sitagliptin possesses anti-inflammatory and anti-apoptotic effects in retinal cells and exerts beneficial effects on the blood-retinal barrier integrity in the retinas of Zucker diabetic fatty rats [31]. Many scholars noted that DPP-4 inhibitors increase the availability of GLP-1 in a range of tissues, and GLP-1 receptors are expressed in the proximal tubules, in the renal glomerulus, and on podocytes. Hyperglycemia can impair GLP-1 action and downregulate GLP-1 receptor expression in the kidneys [32]. In human proximal tubular cells, a GLP-1 receptor agonist may inhibit the advanced glycation end product (AGE)-receptor for AGE (RAGE)-mediated asymmetric dimethylarginine generation via inhibition of reactive oxygen species generation, thereby providing protection against the development and progression of diabetic kidney damage [33]. However, we did not find the effect of protective renal function by sitagliptin in our study.

### Limitations

There are several limitations of our present study. First, our present study was a sub-analysis of the PROLOGUE study, and the number of study subjects was relatively small. Therefore, there is no sample size for a power calculation since fasting glucose, HbA1c, systolic pressure, diastolic pressure, serum urea nitrogen and serum creatinine were voluntary measured parameters in the PROLOGUE trial, and this may be underpowered. Second, the PROLOGUE study was conducted with a PROBE design, which might have introduced bias in the assessment of outcomes. Third, the patients of our study are Japanese, and perhaps race, gene polymorphism, diet and other factors also dictate the effects of sitagliptin in patients with type 2 diabetes complicated with hypertension. Fourth, baseline fasting glucose, HbA1c, systolic pressure, diastolic pressure, serum urea nitrogen and serum creatinine levels were not very high, probably because participants’ diabetes was well-controlled without insulin treatment in the participants. Therefore, the findings of our study should be interpreted with caution.

## MATERIALS AND METHODS

### Study design and patients

The rationale and design of the PROLOGUE study (University Hospital Medical Information Network Center: ID 000004490) have been described previously [34]. The PROLOGUE study was a multicenter, prospective, randomized, open-label trial and blinded-endpoint trial carried out with the participation of 48 Japanese institutions. Eligible patients were at least 30 years of age who had type 2 diabetes with an HbA1c level of 6.2–9.4% despite conventional treatment with diet, exercise and/or pharmacological therapy with oral antihyperglycemic agents (except incretin-related therapy) for more than 3 months. Patients who had taken a DPP-4 inhibitor, such as glucagon-like peptide-1 (GLP-1) analogs, or insulin before randomization were excluded. Other exclusion criteria are described elsewhere.

Between June 2011 and September 2012, a total of 463 patients with type 2 diabetes were enrolled in the PROLOGUE study and randomly assigned in a 1:1 ratio to either add-on sitagliptin treatment (sitagliptin group: n=232) or conventional antihyperglycemic treatment (conventional group: n=231). The treatment randomization was based on age, gender, use of statins, pretreatment diabetic type (non-pharmacological or pharmacological treatment), HbA1c level (<7 or ≥7%), office systolic blood pressure (<135 or ≥135 mm Hg), and maximum IMT (<1.0 or ≥1.0 mm) [35]. All patients were treated with the aim of achieving a targeted HbA1c level of less than 6.2% or a fasting plasma glucose level of less than 110 mg/dL during the study period. Treatment of patients in the sitagliptin group initially began with sitagliptin at a dose of 50 mg daily. If further glycemic intervention was necessary, the dose of sitagliptin was increased up to 100 mg daily within 3 months, and conventional antihyperglycemic agents, other than DPP-4 inhibitors, GLP-1 analogs and/or insulin, were added. If further glycemic intervention was necessary in patients in the conventional group, antihyperglycemic agents, other than DPP-4 inhibitors, GLP-1 analogs and/or insulin, were added. All of the patients were followed up annually for 2 years until September 2014.

In the PROLOGUE study, the primary endpoint was the change in mean common carotid artery IMT at 24 months after treatment. Carotid ultrasound examinations were performed at the beginning of treatment and after 12 and 24 months of treatment. The secondary outcomes included changes in FMD in the brachial artery after 12 and 24 months of treatment. In all of the participating institutions, FBS, HbA1c, BP, DP, BUN and SCR were measured in all patients during an optional examination at the beginning of the study and after 12 and 24 months of treatment. Among the 463 participants in the PROLOGUE study, 365 patients were diagnosed with type 2 diabetes mellitus complicated with hypertension. One hundred and eighty-nine patients were randomly assigned to either add-on sitagliptin treatment (sitagliptin group) and 176 patients were randomly assigned to continue conventional treatment (conventional group). The data for these 365 patients were analyzed in our study. This sub-study is a pre-specified analysis. The ethical committees of the participating institutions approved the study protocol. Written informed consent for participation in the study was obtained from each subject.

### Study protocol

All studies were performed in the morning, after overnight fasting, in a quiet, dark, and air-conditioned room (constant temperature of 22–25 °C). The subjects were kept in the supine position throughout the study. The observers were blind to the type of treatment.

### Clinical and other measurements

The same protocol for measuring FMD in the brachial artery was used in our study. Height and weight were measured and body mass index (BMI, kg m^−2^) was calculated. At the time of the health examinations, after a brief period of rest, SBP and DBP were measured in either the right or left arm using a sphygmomanometer (Omron Corporation, Kyoto, Japan) with the participant in a sitting position. Blood pressure was measured once in most participants, but up to three measurements were taken at 1–2-min intervals in participants who had hypertensive or prehypertensive SBP and DBP values. The lowest reading was used in the analysis to assess the incidence of hypertension. Smoking habit and parental history of hypertension were assessed by a questionnaire, as was a self-reported history of hypertension. Blood samples were collected after an overnight fast, and measurements were made using an automatic clinical chemistry analyzer (Hitachi, LABOSPECT 008, Tokyo, Japan). FBS, HbA1c, BUN and SCR levels were measured using enzymatic methods at the beginning of the study and after 12 and 24 months of treatment.

### Statistical analysis

Results are presented as the number (percent) or the mean±SD. Categorical variables were compared using the chi-square test. We compared the mean values of continuous variables between the 2 groups using student’s unpaired *t*-test. Differences in the mean values of continuous variables between baseline, 12 months and 24 months were compared using repeated measures analysis of variance.

In the analysis comparing the treatment effects, the baseline-adjusted means and their 95% CIs, as estimated by analysis of covariance (ANCOVA), were compared between the two groups. This analysis was carried out while considering the variation caused by treatment effects. Furthermore, a multivariate regression analysis was conducted for adjusting confounding factors, such as age, body height, body weight, DBP, SBP, creatinine, heart rate, uric acid, total cholesterol, fasting plasma glucose, HDL, blood urea nitrogen, HBA1C, cystatin-C, triglyceride, waist circumferences, small dense LDL, high molecular weight adiponectin, insulin, history of dyslipidemia, history of kidney disease, history of liver disease, history of cerebrovascular disorder, history of cardiovascular disorder, history of cerebral infarction, history of cerebral hemorrhage, history of transient ischemic attacks, history of cerebrovascular disorder, history of myocardial infarction, history of arrhythmia, history of percutaneous coronary intervention, history of heart failure, history of cardiovascular disorder, smoking habit, drinking habit, and history of coronary artery bypass graft.

All analyses were performed using Empower (R) (www.Empowerstats.com, X &Y solutions, inc. Boston MA) and the R Project (http://www.R-project.org).

### Prologue study investigators

The PROLOGUE study is a multicenter collaboration. In addition to the listed authors, the following PROLOGUE Study Investigators were involved in this study: Masayoshi Ajioka (Department of Cardiovascular Internal Medicine, Tosei General Hospital); Toru Aoyama (Cardiology Center, Nagoya Kyoritsu Hospital); Tetsuya Babazono (Department of Medicine, Diabetes Center, Tokyo Women’s Medical University School of Medicine); Yasuko K. Bando (Department of Cardiology, Nagoya University Graduate School of Medicine and National Hospital Organization Nagoya Medical Center); Hiroyuki Daida (Department of Cardiovascular Medicine, Juntendo University Graduate School of Medicine); Jun Fukui (Division of Cardiology, Hokusho Central Hospital); Kumiko Hamano (Department of Diabetes and Endocrinology, Kanto Rosai Hospital); Shigemasa Hashimoto (Department of Cardiology, Karatsu Red Cross Hospital); Kazunori Hayashi (Department of Cardiology, Nakatsugawa Municipal Hospital); Tsutomu Hirano (Department of Diabetes, Metabolism, and Endocrinology, Showa University School of Medicine); Hideki Horibe (Department of Cardiovascular Medicine, Gifu Prefectural Tajimi Hospital); Kazuo Ibaraki (Department of Internal Medicine, Karatsu Red Cross Hospital); Takako Iino (Department of Cardiovascular and Respiratory Medicine, Akita University Graduate School of Medicine); Kenji Iino (Department of Cardiovascular and Respiratory Medicine, Akita University Graduate School of Medicine); Yutaka Ishibashi (Department of General Medicine, Shimane University Faculty of Medicine); Yuko S. Ishiguro (Department of Cardiology, Mitsubishi Nagoya Hospital); Masaharu Ishihara (Division of Cardiovascular Medicine and Coronary Heart Disease, Hyogo College of Medicine); Ryoji Ishiki (Division of Internal Medicine, Toyota Memorial Hospital); Tomoko Ishizu (Department of Clinical Laboratory Medicine, Faculty of Medicine, University of Tsukuba); Hiroshi Ito (Department of Cardiovascular and Respiratory Medicine, Akita University Graduate School of Medicine); Masaaki Ito (Department of Cardiology and Nephrology, Mie University Graduate School of Medicine); Yoshito Iwama (Department of Cardiology, Meijo Hospital); Hideo Izawa (Department of Cardiology, Fujita Health University Banbuntane Hotokukai Hospital); Kohei Kaku (Department of Internal Medicine, Kawasaki Medical School); Haruo Kamiya (Division of Cardiology, Japanese Red Cross Nagoya Daiichi Hospital); Kenshi Kan (Division of Diabetes, Metabolism and Endocrinology, Tokyo Medical University Hospital); Naoki Kashihara (Department of Nephrology and Hypertension, Kawasaki Medical School); Akira Kimura (Department of Cardiology, Meijo Hospital, Federation of National Public Service Personnel Mutual Aid Association); Ichiro Kishimoto (Department of Atherosclerosis and Diabetes, National Cerebral and Cardiovascular Center); Kazuo Kitagawa (Department of Neurology, Tokyo Women’s Medical University); Masafumi Kitakaze (Department of Clinical Medicine and Development, National Cerebral and Cardiovascular Center); Tomoki Kitano (Department of Cardiology, National Hospital Organization Nagoya Medical Center); Yoshihisa Kizaki (Department of Cardiology, Sasebo Chuo Hospital); Kenji Kohara (Division of Diabetes, Endocrinology and Metabolism, Kawasaki Medical School); Hiroshi Koiwaya (Department of Cardiology, Miyazaki Medical Association Hospital); Taizo Kondo (Department of Cardiology, Gifu Prefectural Tajimi Hospital); Toshimitsu Kosaka (Department of Cardiovascular and Respiratory Medicine, Akita University Graduate School of Medicine); Nehiro Kuriyama (Department of Cardiology, Miyazaki Medical Association Hospital); Shigetaka Kuroki (Eguchi Hospital); Koji Maemura (Department of Cardiovascular Medicine, Graduate School of Biomedical Sciences, Nagasaki University); Hiroaki Masuzaki (Second Department of Medicine, Division of Endocrinology, Diabetes and Metabolism, Hematology, Rheumatology, Graduate School of Medicine, University of the Ryukyus); Munehide Matsuhisa (Diabetes Therapeutics and Research Center, Tokushima University); Kaori Miwa (Department of Neurology and Stroke Center, Osaka University Graduate School of Medicine); Takashi Miwa (Department of Diabetes, Endocrinology, Metabolism and Rheumatology, Tokyo Medical University); Tetsuro Miyazaki (Department of Cardiovascular Medicine, Juntendo University School of Medicine); Kazutaka Mori (Department of Cardiology, Nagoya Medical Center); Tomoatsu Mune (Division of Diabetes, Endocrinology and Metabolism, Kawasaki Medical School); Ikue Nakadaira (Diabetes and Endocrinology, Kanto Rosai Hospital); Mashio Nakamura (Department of Cardiology and Nephrology, Mie University Graduate School of Medicine); Yoshihito Nakashima (Department of Cardiovascular Disease, Tosei General Hospital); Masayuki Nakayama (JCHO Saga Central Hospital); Mamoru Nanasato (Cardiovascular Center, Japanese Red Cross Nagoya Daini Hospital); Kosaku Nitta (Department of Medicine, Kidney Center, Tokyo Women’s Medical University); Yasunori Oguma (Department of Cardiovascular and Respiratory Medicine, Akita University Graduate School of Medicine); Hirotoshi Ohmura (Department of Cardiovascular Medicine, Juntendo University Graduate School of Medicine); Shinji Okubo (Japan Labour Health and Welfare Organization, Kashima Hospital, Special Department and Cardiology, Tokyo Medical University); Jun-ichi Oyama (Department of Cardiovascular Medicine, Saga University); Sosho Ri (Division of Diabetes, Metabolism and Endocrinology, Internal Medicine Center, Showa University Koto Toyosu Hospital); Kenji Sadamatsu (Department of Cardiology, Saga Medical Center Koseikan); Makoto Saitoh (Department of Internal Medicine, Nishio Municipal Hospital); Masaki Sakakibara (Department of Cardiology, Handa City Hospital); Yasunori Sato (Department of Global Clinical Research, Graduate School of Medicine, Chiba University); Yoshisato Shibata (Department of Cardiology, Miyazaki Medical Association Hospital); Toshimasa Shigeta (Department of Cardiology, Gifu Prefectural Tajimi Hospital); Kenei Shimada (Department of Internal Medicine and Cardiology, Osaka City University Graduate School of Medicine); Fuji Somura (Department of Cardiology, Nagoya Central Hospital); Takashi Takei (Department of Medicine, Kidney Center, Tokyo Women’s Medical University); Toshiro Tanaka (Department of Internal Medicine, Nishio Municipal Hospital); Yoshito Tanioka (Division of Cardiology, Omura Municipal Hospital); Akihiro Terasawa (Department of Cardiology, Kasugai Municipal Hospital); Masahiko Tsujii (Department of Gastroenterology, Osaka Rosai Hospital); Shuichi Tsuruoka (Department of Nephrology, Nippon Medical School); Hiroyuki Tsutsui (Department of Cardiovascular Medicine, Hokkaido University Graduate School of Medicine); Hisashi Umeda (Division of Cardiology, Toyota Memorial Hospital); Mitsuyoshi Urashima (Division of Molecular Epidemiology, Jikei University School of Medicine); Hiroki Watanabe (Department of Internal Medicine, Nishio Municipal Hospital); Masato Watarai (Cardiovascular Center, Anjo Kosei Hospital); Takaaki Yamada (Department of Cardiology, Nagoya Medical Center); Hiroshi Yamamoto (Cardiovascular Surgery, Yamamoto Memorial Hospital); Akira Yamashina (Department of Cardiology, Tokyo Medical University); Kentaro Yamashita (Department of Cardiology, Nagoya University Graduate School of Medicine and National Hospital Organization Nagoya Medical Center); Takanori Yasu (Department of Cardiovascular Medicine, Dokkyo Medical University Nikko Medical Center); Chie Yasuoka (Department of Cardiovascular Medicine, Omura Municipal Hospital); Kiyoshi Yokoi (Department of Cardiovascular Medicine, Gifu Prefectural Tajimi Hospital).

### Availability of data and materials

All relevant data are within the paper and the Dryad Digital Repository (http://dx.doi.org/10.5061/dryad.qt743/2).

### Ethics approval and consent to participate

The ethical committees of the participating institutions approved the study protocol. All participants provide written informed consent before data collection.

## CONCLUSIONS

In our study, there was no evidence that treatment with sitagliptin can improve fasting glucose, HbA1c, systolic pressure, diastolic pressure or renal function in type 2 diabetes patients complicated with hypertension during a 2-year study period. Thus, sitagliptin may be used in type 2 diabetes patients complicated with hypertension, but with caution. Further randomized trials are warranted.
